# Supplementary figures and images for: Predicting foot orthosis deformation based on its contour kinematics during walking
Source: PLoS One. 2020 May 7;15(5):e0232677. doi: 10.1371/journal.pone.0232677 (PMC7205218; doi:10.1371/journal.pone.0232677)

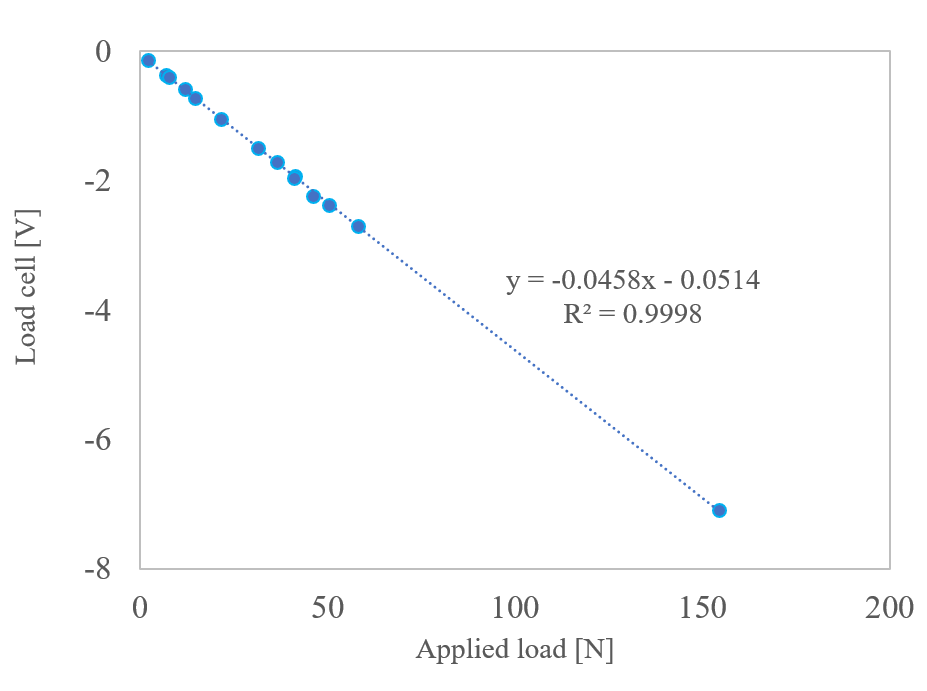

Supplement: S1 Fig — (TIF) [file pone.0232677.s006.tif]

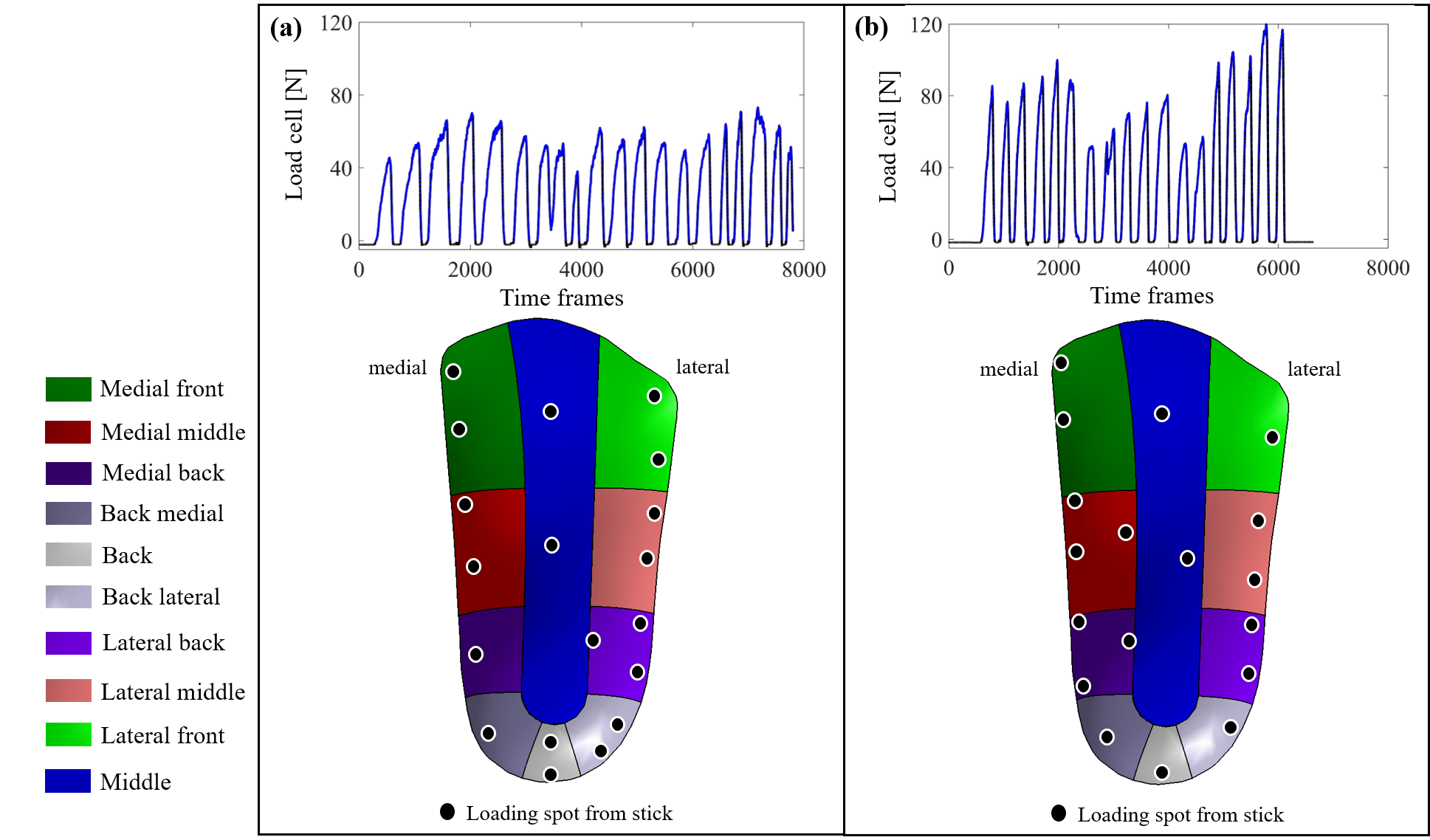

Supplement: S2 Fig — (TIF) [file pone.0232677.s007.tif]
