# Supplementary material for: Predicting foot orthosis deformation based on its contour kinematics during walking
Source: PLoS One. 2020 May 7;15(5):e0232677. doi: 10.1371/journal.pone.0232677 (PMC7205218; doi:10.1371/journal.pone.0232677)
Supplement: S1 Table — (DOCX) [file pone.0232677.s008.docx]

| **Subject** | **Speed** | **Step length [m]** | |
| --- | --- | --- | --- |
| #No. | [m/s] | Sport | Regular |
| 1 | 0.8 | 0.53 | 0.52 |
| 2 | 1.1 | 0.39 | 0.46 |
| 3 | 0.7 | 0.48 | 0.46 |
| 4 | 1.0 | 0.56 | 0.55 |
| 5 | 1.0 | 0.48 | 0.47 |
| 6 | 0.9 | 0.52 | 0.52 |
| 7 | 1.1 | 0.59 | 0.59 |
| 8 | 1.0 | 0.56 | 0.53 |
| 9 | 1.0 | 0.60 | 0.64 |
| 10 | 1.0 | 0.57 | 0.56 |
| 11 | 1.0 | 0.51 | 0.51 |
| 12 | 0.8 | 0.49 | 0.50 |
| 13 | 1.2 | 0.62 | 0.60 |
| **mean ± SD** | 1.0 ± 0.1 | 0.53 ± 0.06 | 0.53 ± 0.05 |
