# Supplementary material for: Predicting foot orthosis deformation based on its contour kinematics during walking
Source: PLoS One. 2020 May 7;15(5):e0232677. doi: 10.1371/journal.pone.0232677 (PMC7205218; doi:10.1371/journal.pone.0232677)
Supplement: Attachment — Submitted filename: Response to Reviewers.docx [file pone.0232677.s009.docx]

**Journal Requirements:**

When submitting your revision, we need you to address these additional requirements:

1. Please ensure that your manuscript meets PLOS ONE's style requirements, including those for file naming. The PLOS ONE style templates can be found at http://www.plosone.org/attachments/PLOSOne_formatting_sample_main_body.pdf and http://www.plosone.org/attachments/PLOSOne_formatting_sample_title_authors_affiliations.pdf

**Answer**: The title page and manuscript was adapted based on the style templates. In title page, the address of corresponding author was removed, and the e-mail address was just kept. In the manuscript, bold type was used for the titles of figures and supporting information

2. Thank you for stating the following in the Competing Interests/Financial Disclosure* (delete as necessary) section:

"The authors have declared that no competing interests exist."

We note that one or more of the authors are employed by a commercial company: Caboma, Montréal.

2.1. Please provide an amended Funding Statement declaring this commercial affiliation, as well as a statement regarding the Role of Funders in your study. If the funding organization did not play a role in the study design, data collection and analysis, decision to publish, or preparation of the manuscript and only provided financial support in the form of authors' salaries and/or research materials, please review your statements relating to the author contributions, and ensure you have specifically and accurately indicated the role(s) that these authors had in your study. You can update author roles in the Author Contributions section of the online submission form.

Please also include the following statement within your amended Funding Statement.

“The funder provided support in the form of salaries for authors [insert relevant initials], but did not have any additional role in the study design, data collection and analysis, decision to publish, or preparation of the manuscript. The specific roles of these authors are articulated in the ‘author contributions’ section.”

If your commercial affiliation did play a role in your study, please state and explain this role within your updated Funding Statement.

**Answer**: Based on the journal suggestion, an amended funding statement is provided here to clarify the role of our funders in this study. Consequently, the author roles in the author contribution was also updated.

This manuscript is a part of research project which is financially supported by NSERC (Natural Sciences and Engineering Research Council) R&D Coop with industrial partners Medicus and Caboma, and MEDTEQ under grant number RDCPJ 506194-16, principal researcher: Mickaël Begon (MB), original project title: "FOOT¡ (Functional Optimized Orthotic Trabecular Insole) :Une orthèse plantaire personnalisée selon la dynamique du pied pour l'impresson 3D" (“customized foot orthosis to meet the demands of foot dynamics for 3D printing”), and URL funder: https://www.nserc-crsng.gc.ca/index_eng.asp.

In addition, FRQNT (The Fonds de recherche du Québec - Nature et technologies) provided financial support in the form of doctoral research scholarships program for foreign students (DE), File No. 208405, awarded to Maryam Hajizadeh (MH), and URL funder: http://www.frqnt.gouv.qc.ca/en/accueil

The funders, either NSERC, MEDTEQ or FRQNT, as well as our industrial partner Medicus did not have any role in the study design, data collection and analysis, decision to publish, or preparation of this manuscript. However, Jean-Philippe Carmona (JPC), a mechanical engineer in Caboma, was involved during our regular meetings and brought technical ideas to improve the algorithms and interpretation of results. JPC also contributed to reviewing and editing the paper.

**Updated Author Contributions:**

Conceptualization: Maryam Hajizadeh, Benjamin Michaud, Mickaël Begon

Data curation: Maryam Hajizadeh, Benjamin Michaud

Formal analysis: Maryam Hajizadeh, Benjamin Michaud

Funding acquisition: Mickaël Begon

Investigation: Maryam Hajizadeh, Benjamin Michaud, Gauthier Desmyttere, Jean-Philippe Carmona

Methods: Maryam Hajizadeh, Benjamin Michaud, Gauthier Desmyttere, Jean-Philippe Carmona

Project administration: Mickaël Begon

Resources: Mickaël Begon, Jean-Philippe Carmona

Supervision: Mickaël Begon

Validation: Maryam Hajizadeh, Gauthier Desmyttere, Jean-Philippe Carmona

Visualization: Maryam Hajizadeh, Benjamin Michaud, Gauthier Desmyttere

Writing – original draft: Maryam Hajizadeh

Writing – review & editing: Maryam Hajizadeh, Benjamin Michaud, Gauthier Desmyttere, Jean-Philippe Carmona, Mickaël Begon

2.2. Please also provide an updated Competing Interests Statement declaring this commercial affiliation along with any other relevant declarations relating to employment, consultancy, patents, products in development, or marketed products, etc.

Within your Competing Interests Statement, please confirm that this commercial affiliation does not alter your adherence to all PLOS ONE policies on sharing data and materials by including the following statement: "This does not alter our adherence to PLOS ONE policies on sharing data and materials.” (as detailed online in our guide for authors http://journals.plos.org/plosone/s/competing-interests). If this adherence statement is not accurate and there are restrictions on sharing of data and/or materials, please state these. Please note that we cannot proceed with consideration of your article until this information has been declared.

Please include both an updated Funding Statement and Competing Interests Statement in your cover letter. We will change the online submission form on your behalf.

Please know it is PLOS ONE policy for corresponding authors to declare, on behalf of all authors, all potential competing interests for the purposes of transparency. PLOS defines a competing interest as anything that interferes with, or could reasonably be perceived as interfering with, the full and objective presentation, peer review, editorial decision-making, or publication of research or non-research articles submitted to one of the journals. Competing interests can be financial or non-financial, professional, or personal. Competing interests can arise in relationship to an organization or another person. Please follow this link to our website for more details on competing interests: http://journals.plos.org/plosone/s/competing-interests.

**Answer**: The statement for competing of interest was modified as following to reflect more details:

“It should also be added that our innovative approach for quantifying the deformation of foot orthosis during walking does not provide any commercial asset at this point. Therefore, the materials presented in this manuscript is not related to any kind of consultancy, patents, products in development, or marketed products. Furthermore, our commercial affiliation does not alter our adherence to PLOS ONE policies on sharing data and materials.

Finally, we confirm that there is no competing of interest, but a fruitful collaboration between our research team and our industrial partners, Caboma and Medicus. On-going studies involving flat feet patients may provide commercial advantages.

3. We note that you have indicated that data from this study are available upon request. PLOS only allows data to be available upon request if there are legal or ethical restrictions on sharing data publicly. For more information on unacceptable data access restrictions, please see http://journals.plos.org/plosone/s/data-availability#loc-unacceptable-data-access-restrictions.

In your revised cover letter, please address the following prompts:

a) If there are ethical or legal restrictions on sharing a de-identified data set, please explain them in detail (e.g., data contain potentially sensitive information, data are owned by a third-party organization, etc.) and who has imposed them (e.g., an ethics committee). Please also provide contact information for a data access committee, ethics committee, or other institutional body to which data requests may be sent.

b) If there are no restrictions, please upload the minimal anonymized data set necessary to replicate your study findings as either Supporting Information files or to a stable, public repository and provide us with the relevant URLs, DOIs, or accession numbers. For a list of acceptable repositories, please see http://journals.plos.org/plosone/s/data-availability#loc-recommended-repositories.

We will update your Data Availability statement on your behalf to reflect the information you provide.

**Answer**: We appreciate the PLOS One policy about sharing the data set with other researchers. We do not have any ethical or legal restrictions on sharing the data set relating to this manuscript. The data set that will be shared is as following:

(1) **S2 File**: The raw data set for the position of markers during training session of sport foot orthosis. The data include 3 sheets namely “Triad orientation”, “Triad position”, and “FO markers”. “Triad position” represents the position of each triad during each time frame of training session. “Triad orientation” that was calculated as the orientation of each triad relative to back triad as explained in Methods section. “FO markers” shows the position of markers on the plantar surface of foot orthosis (55 markers) during each time frame of training session. The developed neural network was trained to find the dependency between triad orientation and the position of FO markers.
(2) **S3 File**: The raw data set for subject 1 during walking with sport foot orthosis. This data also included two sheets namely “Triad orientation” and “Triad position”. The data in “Triad orientation” is used to predict the position of markers on the plantar surface of foot orthosis during walking.
(3) **S4 File and S5 File**: The result files that represent the displacement of sport foot orthosis and regular foot orthosis respectively during walking for each included subject. The results include “DispEachPoint” and “DispEachPointMean” which shows the displacement of each predicted marker on foot orthosis plantar surface during stance phase of walking relative to its corresponding position in static non weight-bearing for each trial and the average of trials respectively. “loc_stance” and “loc_meanstance” show the location of each predicted marker during stance phase of walking. “peaks” and “peaksMean” represent the minimum (depression) and maximum (reformation) value of displacement during walking.

**Reviewers' comments:**

**Reviewer's Responses to Questions**

Comments to the Author

1. Is the manuscript technically sound, and do the data support the conclusions?

Reviewer #1: Yes

**Answer**: Many thanks for your kind consideration and for the time you assigned for reviewing our manuscript.

2. Has the statistical analysis been performed appropriately and rigorously?

Reviewer #1: Yes

3. Have the authors made all data underlying the findings in their manuscript fully available?

Reviewer #1: Yes

4. Is the manuscript presented in an intelligible fashion and written in standard English?

Reviewer #1: Yes

**5. Review Comments to the Author**

Reviewer #1: A good paper, well written with a good hypothesis on a important subject.

My main concerns are following six:

1) Why did you select the comfortable speed in walking?

Walking speed influenced on the force for deformation of FO. Are walking speed and step length same in each conditions?

**Answer:** Based on the protocol of this study, within-subject speed was the same, where between-subject speed could be different depending on each participant comfortable pace. The results of previous literature were considered to choose this option for designing our protocol. Previous research showed that at speeds either slower or faster than preferred speed the variability increases in young adults during treadmill walking [1]. In addition, it has been observed that each individual chooses a comfortable speed at which he reduces the energy consumption by parallelizing the passive mechanical properties of the legs with muscle activation. More stable condition has also been attributed to preferred walking speed [2-5]. On the other hand, some studies have addressed changes in gait pattern as an effect of walking speed [6]. However, as paired t-test is used in this study to look at the effect of foot orthosis rigidity, we believe that within-subject variability plays more important rule than between-subject differences for the purpose of this study.

The following sentences were added to manuscript for more clarification:

“Each participant was asked to walk on a treadmill for 5 minutes for habituation, where his comfortable speed was acquired for the following measurements. Then, the participant walked for 3 minutes at this acquired speed for each sport and regular FO condition. A rest period of 5 minutes was given between conditions to avoid fatigue effects. The last 30 s of each trial was used for further processing.” [lines 161-168]

In addition, the speed of walking and step length for each subject was added to the supporting information (S1 Table) as following:

| **Subject** | **Speed** | **Step length [m]** | |
| --- | --- | --- | --- |
| #No. | [m/s] | Sport | Regular |
| 1 | 0.8 | 0.53 | 0.52 |
| 2 | 1.1 | 0.39 | 0.46 |
| 3 | 0.7 | 0.48 | 0.46 |
| 4 | 1.0 | 0.56 | 0.55 |
| 5 | 1.0 | 0.48 | 0.47 |
| 6 | 0.9 | 0.52 | 0.52 |
| 7 | 1.1 | 0.59 | 0.59 |
| 8 | 1.0 | 0.56 | 0.53 |
| 9 | 1.0 | 0.60 | 0.64 |
| 10 | 1.0 | 0.57 | 0.56 |
| 11 | 1.0 | 0.51 | 0.51 |
| 12 | 0.8 | 0.49 | 0.50 |
| 13 | 1.2 | 0.62 | 0.60 |
| **mean ± SD** | 1.0 ± 0.1 | 0.53 ± 0.06 | 0.53 ± 0.05 |

2) L 82．Why did you consider to be important for deformation of the FO?

**Answer:** We thank Reviewer #1 for asking this question. To our view, it is important to know FO deformation in order to improve the design of FO based on the biomechanical demands of different foot regions during dynamic activities. The primary requirement to achieve this goal was to validate the prediction of foot orthosis deformation on its whole plantar surface. FO deformation would then be used as a metric that represents the dynamic behavior of foot orthosis design. The next step would be to look at the interaction between foot biomechanics (kinematics and plantar pressure distribution) and the dynamic behavior of FO, i.e. FO deformation. Through this step, we could find out which metrics of foot biomechanics account for variation in FO deformation in each region of FO. Finally, this will help us to adapt the design of FO in each region based on the corresponding foot biomechanics and the level that we are interested to change based on the foot abnormality.

The importance of estimating FO deformation has been better addressed in Introduction [Lines 63-68] and the future implication is pointed out in Discussion [Lines 574-578].

3) L114．Why did you select the normal feet? Low arch feet have larger contact area of the FO than normal feet, and contact area influenced on the deformation of the FO. Moreover, persons using FO have the flat foot deformity.

**Answer**: We appreciate Reviewer #1 for pointing out this important issue. In fact, the main aim of this manuscript was to (a) present and apply our artificial intelligence approach to predict the deformation of foot orthosis, and (b) to see if this technique is capable to differentiate between different rigidities of foot orthosis. It was assumed that healthy subjects with normal foot structure who walk with the same general 3D printed foot orthosis would impose less inter-subject variability compared to flatfoot subjects with abnormal foot posture (pronated foot and low medial arch). Therefore, healthy subjects were targeted for this study to more explicitly focus on the deformation of foot orthosis rather than incorporating the effect of abnormal foot deformity into the FO deformation. In the next step of this project, the validated technique would be implemented on subjects with flat foot deformity to find out their foot orthosis deformation during walking with their customized 3D printed foot orthosis.

4) What definitions as the normal feet? Asymptomatic? No deformation?

**Answer:** The inclusion criteria for participants was to be free from any limb injuries at the time of testing and having no known history of foot structural abnormalities or pathologies. The subjects were asked whether they have ever used foot orthosis or therapeutic insoles for any reason of pain or foot injuries especially flatfoot deformity. In addition, two observers, GD and MH, had to examine and include the subjects with normal medial arch during weight bearing/non weight bearing position and normal rearfoot orientation relative to tibia long axis.

These sentences were added to the Methods section [lines 116-124].

5) How did you consider the bending the sole (outsole) of the shoe during walking? I think you estimate the deformation of the FO, however your methods estimate the deformation of the FO and outsole.

**Answer:** This is a good question to be brought up. The triad markers were attached to FO contour, and they did not have any contact with the shoe sole. As a result, we believe that what is measured in this study is the deformation of the FO and not a combination of shoe sole and FO deformation. However, it is true that FO is in direct contact with both foot and shoe sole. Therefore, the deformation of FO is affected by the loading from foot as well as the boundary conditions imposed from shoe sole. It means that depending on the shape and mechanical properties of the shoe sole, we would change the boundary condition and the degree of freedom for the range of deformation on the plantar surface of FO. For example, a flexible shoe sole might provide more freedom for the vertical displacement of FO where a rigid FO might limit its displacement. In addition, a flat shoe sole provides different degree of freedom for the FO compared to a shoe sole with contour shape. The following sentences were added to the discussion to address this issue:

“As foot orthosis is in direct contact with both foot and shoe sole, the deformation of FO is affected by the loading from foot and the boundary conditions imposed from shoe sole. It means that depending on the shape and mechanical properties of the shoe sole, we would change the degree of freedom for the range of deformation on the plantar surface of FO. In order to modulate the effect of shoe sole on the variability of FO deformation between subjects, standard shoes (New Balance 860 v8) were used for all subjects during walking. However, it is necessary to address the difference in the effect of shoe sole during training versus walking session as a limitation of this study. In the training session, the FO was fitted on a wooden plate covered with a soft material corresponding to a shoe midsole property. The movement of the shoe sole was therefore constrained in the training session. In contrast, the shoe sole during walking was capable to move and deform. This would lead to differences in the boundary condition applied from shoe sole on the deformation of FO. This limitation might be figured out in future studies by improving the setup design.” [lines 550-566]

6) Why did you select to compress using stick? When weight loaded on the FO, FO did not apply the compression force in “point”. The deformation of the FO is influenced by how to applying the force. Can we assume that the difference in the way applying force has no effect?

**Answer**: Thanks for pointing out to this limitation of our study, which is addressed in the discussion section [lines 531-538]. A 6-mm diameter stick was used in this study to apply point forces during the training session rather than the real distributed force that exist during walking. During training session, 55 retroreflective markers were taped on the small plantar surface of foot orthosis. The load application tool to deform the FO had to stimulate the loads during walking, in addition to not hiding the markers from the view of VICON cameras, which was the main challenge during data collection. For example, designing a wider stick with multiple tips (like a brushing hair) could stimulate the distributed load, in the cost of missing the position of a lot of markers.

However, some points were considered in this study to make up for this limitation. During data collection, several repetitive loads were applied on different regions of FO using the stick which was equipped to a load cell for capturing the applied forces as well as retroreflective markers to retrieve the location of loading on FO (S2 Fig). This enabled us to check whether loading during the training session replicates what happens during walking. Although we cannot assume that the way of applying load has no effect, we showed that the range of loading that was applied manually from stick to the FO during training session could cover the range of pressure that all subjects applied to each 10 regions of FO during walking for both FOs (Fig 3).

Nevertheless, this is a good contribute to the assessment of this "gray" clinical important of foot and ankle treatment.

6. PLOS authors have the option to publish the peer review history of their article (what does this mean?). If published, this will include your full peer review and any attached files.

Reviewer #1: No

**References:**

1.	Kang, H.G., J.B.J.G. Dingwell, and posture, *Separating the effects of age and walking speed on gait variability.* 2008. **27**(4): p. 572-577.

2.	Holt, K.G., J. Hamill, and R.O.J.H.M.S. Andres, *The force-driven harmonic oscillator as a model for human locomotion.* 1990. **9**(1): p. 55-68.

3.	Holt, K.G., et al., *Predicting the minimal energy costs of human walking.* 1991. **23**(4): p. 491-498.

4.	Jordan, K., et al., *Walking speed influences on gait cycle variability.* 2007. **26**(1): p. 128-134.

5.	Margaria, R., *Biomechanics and energetics of muscular exercise*. 1976: Oxford University Press, USA.

6.	Fukuchi, C.A., R.K. Fukuchi, and M.J.S.r. Duarte, *Effects of walking speed on gait biomechanics in healthy participants: a systematic review and meta-analysis.* 2019. **8**(1): p. 153.
